# Recent advances in combating Nipah virus

**DOI:** 10.12703/r/10-74

**Published:** 2021-09-29

**Authors:** Kendra Johnson, Michelle Vu, Alexander N Freiberg

**Affiliations:** 1 Department of Microbiology and Immunology, University of Texas Medical Branch, Galveston, Texas, USA; 2 Department of Pathology, University of Texas Medical Branch, Galveston, Texas, USA

**Keywords:** Nipah virus, henipavirus, respiratory disease, encephalitis, antivirals, antibodies

## Abstract

Over the past 20 years, Nipah virus (NiV) has emerged as a significant, highly pathogenic bat-borne paramyxovirus causing severe respiratory disease and encephalitis in humans, and human-to-human transmission has been demonstrated in multiple outbreaks. In addition to causing serious illness in humans, NiV is a zoonotic pathogen capable of infecting a wide range of other mammalian species, including pigs and horses. While NiV has caused less than 700 human cases since its discovery in 1998/1999, the involvement of intermediate agricultural hosts can result in significant economic consequences. Owing to the severity of disease, capacity for human-to-human transmission, zoonotic potential, and lack of available approved therapeutic treatment options, NiV has been listed by the World Health Organization in their Blueprint list of priority pathogens as one of the eight most dangerous pathogens to monitor and prepare countermeasures to prevent a pandemic. Here, we discuss progress towards the development of therapeutic measures for the treatment of NiV infection and disease.

## Introduction

Nipah virus (NiV), and the related Hendra virus (HeV), are highly pathogenic, emerging zoonotic bat-borne RNA viruses belonging to the henipavirus (HNV) genus within the *Paramyxoviridae* family. NiV was first identified in 1998/1999 in Malaysia in an outbreak of encephalitis in pig farmers^1,2^, resulting in the culling of more than 1 million pigs, which contributed to controlling the outbreak but ultimately had a significant economic impact^3^. Subsequently, NiV has caused nearly annual outbreaks in Bangladesh and India^2,4–9^. Two genetically divergent strains of NiV have been identified, NiV Malaysia (NiV-M) and NiV Bangladesh (NiV-B), with the latter being the currently circulating strain^10^. In humans, infection with NiV is associated with severe, acute respiratory illness, as well as acute, relapsed, or late-onset encephalitis, and case fatality rates range from 40 to 100%^11,12^. Treatment of patients is mainly reliant on supportive care, with maintenance of airways, breathing, and circulation as well as fluid and electrolyte balance. To date, only an agricultural subunit vaccine for the related HeV is licensed^13^. While primarily associated with spillover events from the natural fruit bat reservoir host, frequent human-to-human transmission has been reported^4,14,15^. Because of the extreme pathogenicity and pandemic potential of NiV, the lack of approved human treatments and vaccines, and its potential for use in (agro)bioterrorism, it is crucial to develop vaccines and therapeutics for NiV^3,16^. This review outlines recent advances in the development of therapeutics and treatments for NiV infection.

## Monoclonal antibodies

Currently, the human cross-reactive monoclonal antibody (mAb) m102.4 is the most promising monoclonal antibody therapeutic treatment for NiV infection. This antibody was affinity matured to strongly neutralize both NiV and HeV attachment glycoprotein G by blocking the interaction of G with the host cellular entry receptors Ephrin B2 and B3^17,18^. m102.4 has demonstrated protection against HNV infection in both ferret and non-human primate (NHP) models of infection. A single intravenous (IV) infusion of m102.4 10 hours after intranasal infection with NiV afforded full protection in a ferret model of disease^19^. Post-exposure studies in the African Green Monkey (AGM) model were even more promising. Here, m102.4 was fully protective in AGMs when treatment was initiated up to 3 days post-infection with HeV and 5 days post-infection with NiV-M, even after onset of clinical symptoms and viremia^20,21^. In both studies, a second dose was administered 2 days after the initial one. Interestingly, a study comparing pathogenicity of NiV-M and NiV-B in the AGM model indicated that the treatment window for NiV-B may be shorter as compared to NiV-M. Commensurate with an accelerated onset of severe disease in NiV-B compared to NiV-M, m102.4 was protective only when administered up to 3 days post-infection with NiV-B. Infected animals receiving initial treatment at 5 days post-infection succumbed to disease^22^.

Results from these studies justified the usage of m102.4 in humans for compassionate use as well as a phase I clinical trial. To date, m102.4 has been administered 14 times for compassionate therapy following high-risk exposure to HNVs^23,24^. No treatment-related adverse effects were reported in any of these cases. Additionally, no recipients of the antibody developed disease, although it is impossible to determine whether this was related to m102.4 treatment. The combination of compassionate therapy for post-exposure treatment in patients and the promising preclinical data from animal studies led to the assessment of safety, tolerability, and immunogenicity of m102.4 in healthy adults in a phase I clinical trial^23^. This study found that the dosages tested were safe and well tolerated. Also, no serious adverse effects resulting in participant withdrawal were reported. Pharmacokinetics evaluations concluded that m102.4 remained active at levels capable of virus neutralization for at least 8 days post-administration. Immunogenicity tests found that no anti-m102.4 antibodies were generated. Although this trial was small (30 participants) and unable to evaluate protective efficacy, the safety and tolerability demonstrated in this study make m102.4 one of the most promising therapeutic options for the treatment of patients with HNV exposure.

Another potential antibody therapy currently under investigation is h5B3.1, a humanized, cross-reactive, neutralizing mAb that targets the fusion glycoprotein F of NiV and HeV, blocking the conformational change required to facilitate membrane fusion and virus infection^25,26^. Previous studies had demonstrated protective efficacy of anti-F mouse polyclonal antibodies or mAbs against NiV and HeV challenge of hamsters, supporting development of the humanized h5B3.1 mAb as a potential therapeutic for use in humans^27,28^. Intraperitoneal administration of h5B3.1 at days 1 and 3, as well as 3 and 5 post-infection, demonstrated protective efficacy against lethal challenges with either NiV or HeV in ferrets. The authors of the study also proposed a combination treatment of h5B3.1 and m102.4 antibodies, targeting both viral surface glycoproteins, as a therapeutic strategy moving forward to minimize the chances of the emergence of escape mutants. However, as *in vivo* characterization of h5B3.1 is currently limited, more studies are required before potential introduction in human patients alone or in combination with m102.4.

## Antiviral drugs

### In humans

The only therapeutic option that has been utilized clinically in NiV patients is ribavirin, a broad-spectrum nucleoside analogue^2,29,30^. Ribavirin is currently licensed for the treatment of respiratory syncytial virus infection, hepatitis C, and viral hemorrhagic fevers and is included on the WHO essential medicines list^31–34^. During the initial 1998/1999 outbreak in Malaysia, an open-label trial of ribavirin was conducted in which 140 patients received treatment. Patients treated prior to the trial or who refused ribavirin treatment served as the control group (n = 54)^29^. In this study, administration of ribavirin was associated with a 36% reduction in mortality and fewer neurological deficits in survivors. In an earlier description of the clinical features presented during that outbreak, it was stated that there appeared to be no significant difference in outcome with ribavirin treatment^2^. More recently, ribavirin was utilized during the 2018 outbreak in Kerala, India, which consisted of a total of 23 cases and only two survivors^4,30^. Both survivors had received oral ribavirin, out of a subset of six patients who received ribavirin therapy^30,35,36^. This corresponds to a 20% reduction in mortality, compared to the 100% mortality observed in a group of six patients not receiving ribavirin. However, the sample size was too small to draw a conclusion on the efficacy of ribavirin against NiV. Ribavirin was also administered as a post-exposure prophylactic to eight healthcare workers who were exposed to infected patients without sufficient PPE during the 2018 outbreak^30^. None developed NiV disease; however, most experienced mild to moderate adverse side effects from the treatment. Overall, ribavirin’s efficacy in the 2018 Kerala outbreak was inconclusive. Moreover, studies of NiV infection in animal models were not promising. Ribavirin administered either alone or in combination with chloroquine (an antimalarial drug) was not protective in the hamster model^37,38^.

### In animal models

No other therapeutics have been utilized for the treatment of NiV infection in patients, and only a few others have been evaluated in animal models. Remdesivir (GS-5734; Veklury^®^), a nucleotide analog that has demonstrated broad-spectrum antiviral activity against filoviruses, paramyxoviruses, and coronaviruses, is one of them^39–41^. In a lethal challenge AGM model for NiV-B, remdesivir led to 100% survival when intravenously administered daily starting 24 hours after infection and continued for 12 days^41^. Two out of the total four NHPs involved in the study developed only mild respiratory signs of disease that resolved by day 14 post-infection. At termination of the study at 92 days post-infection, viral RNA was found in the brain of one animal. While these results are highly encouraging, future studies need to be performed to further evaluate remdesivir’s antiviral efficacy. Remdesivir was also recently included in a clinical trial evaluating Ebola therapeutics in the context of the 2018 Democratic Republic of Congo outbreak^42^. Although this study found it to be less effective against Ebola virus disease compared to mAb treatments, remdesivir did appear to be safe. Recently, remdesivir has been utilized as a compassionate therapy for the treatment of patients with SARS-CoV-2, although the efficacy is still unclear^43–45^. Multiple clinical trials are currently ongoing^46^.

Additional antivirals with promising efficacy have been evaluated for the treatment of NiV infection in small animal models. Favipiravir (T-705; Avigan^®^) is a small molecule purine analog antiviral that is licensed for the management of emerging pandemic influenza infections in Japan^47^. In the Syrian golden hamster model, favipiravir demonstrated full protection against lethal infection with NiV-M when administered immediately after infection and continued daily for 14 days^48^. None of the treated animals developed any clinical signs of disease throughout the course of the study, and no viral RNA or pathological changes in tissues were observed. Future studies need to evaluate the post-exposure antiviral efficacy of favipiravir. Griffithsin (GRFT), a homodimeric high-mannose oligosaccharide-binding lectin, is currently being evaluated in clinical trials as a topical microbicide against human immunodeficiency virus 1 (HIV-1)^49,50^. In cell culture studies, GRFT, as well as a synthetic trimeric tandemer (3mG) and an oxidation-resistant GRFT (Q-GRFT), demonstrated antiviral activity against NiV in the nanomolar range^51^. The prophylactic potential of 3mG and Q-GRFT was evaluated in the Syrian hamster model for NiV-B and resulted in overall survival rates of 15% and 35%, respectively. Future studies are needed to further evaluate and develop Q-GRFT for the treatment of NiV infection.

### *In vitro* 

4'azidocytidine (R1479), a cytidine analog shown to have broad-spectrum antiviral activity against flaviviruses and a pneumovirus, exhibited strong antiviral effects against NiV *in vitro* in the low micromolar range^52–55^. However, balapiravir, the prodrug of R1479, did not show promising results in clinical trials treating flavivirus infections; the trials were discontinued owing to poor prodrug efficacy and negative side effects^53,56,57^. Derivatives of R1479 resulted in more encouraging results compared to R1479. The 2'-monofluoro- and 2'difluoro-modified deriva.tives of R1479 (2'F-4'N3-C and 2'diF-4'N3-C) exhibited up to 20-fold increased antiviral effects on NiV than R1479 *in vitro* and revealed less cytotoxicity^58^. The greater antiviral efficacy and lower cytotoxic effects of 2'F-4'N3-C and 2'diF-4'N3-C make the R1479 derivatives a more promising therapeutic avenue compared with R1479 for future evaluation.

4'-chloromethyl-2'-deoxy-2'-fluorocytidine (ALS-8112) is another antiviral cytidine analog and the parent nucleoside of lumicitabine, which has undergone phase I and phase II clinical trials to treat respiratory syncytial virus infections^59–61^. ALS-8112 displayed strong antiviral effects against NiV *in vitro* in the low micromolar range, with minimal cytotoxic effects in multiple cell lines except for human peripheral blood mononuclear and lymphoblastoid cells^59^. However, caution must be taken in optimizing ALS-8112 dosage to prevent neutropenia and lymphopenia if further evaluated as a potential antiviral against NiV infection in animal models.

In addition to targeting the viral replication machinery, peptide fusion inhibitors aimed at inhibiting viral fusion with the host cellular membrane have been evaluated as well. Optimized lipopeptide fusion inhibitors (with cholesterol or tocopherol conjugated to the polypeptide using dPEG) exhibited protection against lethal NiV infection in Syrian golden hamsters (50% survival) and AGMs (33% survival) after prophylactic administration^62,63^. Enfuvirtide (FuzeonTM) is an FDA-approved analogous therapeutic for HIV-1 and is also a lipopeptide fusion inhibitor which has the potential to move forward as an effective antiviral^64,65^. Future development of potent lipopeptide inhibitors for NiV infection is needed.

One other strategy that has demonstrated potential is the use of defective interfering particles (DIPs), which contain defective genomes that alter the dynamics of a viral population to inhibit NiV replication. *In vitro* assays demonstrate that naturally occurring and *in silico*-designed DIPs decrease viral titer 100-fold and reduce cytopathic effects in Vero cells^66^. While currently exploratory as a form of treatment for NiV, DIPs should undergo future animal studies. Studies using DIPs for influenza A virus have been promising^67–70^.

## Discussion

NiV remains a pathogen of significant concern because of its high pathogenicity, demonstrated potential for human-to-human transmission, and lack of approved treatments. From the range of antivirals tested so far against infectious NiV, only two have demonstrated therapeutic efficacy in the NHP model: the m102.4 monoclonal antibody and the nucleotide analogue remdesivir. m102.4 is the only potential therapeutic that has been evaluated in humans specifically for the treatment of NiV. Currently, m102.4 is perhaps the most promising therapeutic option from an efficacy standpoint; however, the requirement for cold chain storage and the intravenous route of administration may not make it the most practical for use in field outbreaks. Ribavirin has been utilized as a compassionate therapy and a post-exposure prophylactic in humans, but its efficacy remains unclear. Small molecule antivirals, specifically nucleoside analogues, have demonstrated good efficacy in blocking NiV infection and need to be further evaluated for their therapeutic potential. However, evaluation of antiviral efficacy against NiV in patients is complicated by the infrequency of cases, and alternative pathways to licensure may be necessary. As many of the currently characterized antiviral treatment candidates appear to have a limited window for treatment efficacy in animal models, timely diagnosis and initiation of treatment will be crucial. Additionally, combinatorial therapy of mAbs and small molecule antivirals might be an effective treatment strategy, as well as the development and approval of a vaccine for NiV and the prevention of spillover events. In addition to the antiviral therapeutics discussed in this review, four vaccine candidates that demonstrated effectiveness in disease-relevant animal models have received funding through the Coalition of Epidemic Preparedness Innovation (CEPI) to be further evaluated in phase I and II clinical trials^71^. These include a HeV glycoprotein subunit vaccine for the prevention of NiV infection, which is currently in phase I clinical trials^72,73^, and three recombinant viral vector vaccines, which are in preclinical development^74–77^. Additionally, with the recent rise to prominence of mRNA vaccines during the SARS-CoV-2 pandemic, this technology may be useful in the future as a potential strategy for NiV vaccines. In one study, a single dose of a lipid nanoparticle nucleoside-modified messenger RNA vaccine encoding the soluble HeV glycoprotein protected up to 70% of Syrian hamsters from lethal NiV challenge, indicating the promise of this particular platform for future prevention of NiV disease^78^.

It should also be noted that a large number of novel HNVs and henipa-like viruses have recently been identified. The expanding diversity of HNVs raises some questions about preparedness for the potential for new spillovers and the emergence of new pathogenic HNVs, which may be important to consider in the context of therapeutic and vaccine development. While m102.4 and h5B3.1 demonstrate cross-protection for pathogenic HNVs, NiV and HeV, it has recently been reported that antibodies elicited by G proteins of NiV and HeV have very limited cross-reactivity and no cross-protection for new members Mojiang virus (MojV) and Ghana virus (GhV)^79^. This study proposed a fusion protein strategy containing epitopes from NiV, HeV, MojV, and GhV as a broad-spectrum vaccine that elicited cross-protection against all four viruses. It may be important to identify strategies for the generation of pan-HNV treatments in the event of the emergence of new pathogenic HNVs.
